# Clinical study on the status of transient thyrotoxicosis after surgery for secondary hyperparathyroidism patients with end-stage renal disease and normal thyroid function

**DOI:** 10.1186/s40001-020-00405-6

**Published:** 2020-03-17

**Authors:** Bao-shan Zou, Jia-shuo Liu, Hong Li, Zhou Xu, Hao Li, Hong-yuan Li, Kai-nan Wu, Ling-quan Kong

**Affiliations:** Department of Endocrine and Breast Surgery, The First Affiliated Hospital of Chongqing Medical University, No.1 You Yi Rd, Chongqing, 400016 China

**Keywords:** Chronic kidney disease, End-stage renal disease, Secondary hyperparathyroidism, Thyrotoxicosis, Parathyroidectomy

## Abstract

**Objective:**

Secondary hyperparathyroidism (SHPT) is a common complication of end-stage renal disease (ESRD), and part of SHPT patients need receive parathyroidectomy (PTX). However, as an important postoperative complication of SHPT, thyrotoxicosis has received little attention. Therefore, in this article, we aimed to study the status of transient thyrotoxicosis after PTX for SHPT patients with ESRD and normal thyroid function.

**Methods:**

A total of 24 SHPT patients with preoperative normal thyroid function, normal thyroglobulin (Tg) and normal thyroid antibodies receiving PTX were enrolled from the Department of Endocrine and Breast Surgery, the First Affiliated Hospital of Chongqing Medical University, from January 2017 to January 2019. Tg, high sensitivity thyrotropin stimulating hormone (sTSH), triiodothyronine (T3), free triiodothyronine (fT3), thyroxine (T4) and free thyroxine (fT4) were evaluated the day before PTX and on day 1, 3 and 5 after PTX. Besides, all enrolled patients were evaluated whether there are symptoms associated with thyrotoxicosis.

**Results:**

Among the 24 SHPT patients, 1 case (4.2%), 8 cases (33.3%) and 13 cases (54.2%) had suffered thyrotoxicosis at the first, third and fifth day after surgery, respectively. Serum FT4 level increased significantly from pre-operation (0.68 ± 0.15 ng/dl, normal range 0.59–1.25 ng/dl) to the third day after operation (1.91 ± 0.97 ng/dl, *p*<0.001) and then gradually decline. The frequencies of serum sTSH lower than the normal level gradually increased from the first day (8.3%) to fifth day (66.7%) after surgery.

**Conclusion:**

Transient thyrotoxicosis is a common postoperative complication of parathyroidectomy for SHPT patients with ESRD and normal thyroid function, and it is necessary for clinicians to evaluate the perioperative thyroid function to make early diagnosis and appropriate prevention and treatment of thyrotoxicosis.

## Introduction

The prevalence of chronic kidney disease (CKD) has reached 8%–16% worldwide and the prevalence of end-stage renal disease (ESRD) is highly unacceptable, especially in developing countries [1, 2]. The incidence of ESRD in developing countries is about 150 per million population [3]. Hemodialysis is a necessary conservative treatment way for ESRD [4]. Secondary hyperparathyroidism (SHPT), as the common complication of ESRD, has increased steadily correspondingly [5]. SHPT occurs as a result of a series of abnormalities in mineral metabolism that cause an increase in parathyroid hormone (PTH) secretion [6]. The main abnormalities include phosphate retention, low calcium, and 1,25-dihydroxyvitamin deficiency. The most common signs and symptoms of SHPT include hypercalcemia, hyperphosphatemia, bone and joint pain and/or fractures, proximal muscle weakness, extraskeletal calcification and/or calciphylaxis and pruritus [7]. Approximately 10% of patients with ESRD need to receive parathyroidectomy (PTX) for SHPT patients [8]. Normally, clinicians are more concerned with recurrent laryngeal nerve damage, hemorrhage, infections, arrhythmia, and electrolyte disturbances after parathyroidectomy, such as hypocalcemia, hyperkalemia, hypophosphatemia, and hypomagnesemia [9]. However, as an important postoperative complication of secondary hyperparathyroidism, transient thyrotoxicosis is often ignored by clinicians. Thyrotoxicosis is a common disease caused by excessive circulating thyroid hormone. Symptoms of overt thyrotoxicosis include sweating, polydipsia, heat intolerance, tremor, nervousness, anxiety, fatigue, palpitations, dyspnoea, nausea and vomiting [10]. Only a few studies have focused on thyrotoxicosis as a result of PTX in patients with SHPT [11, 12]. We aimed to identify the status of transient thyrotoxicosis and thyroxine fluctuations after PTX for SHPT patients with ESRD and normal thyroid function.

## Methods

### Patients

A total of 24 SHPT patients with preoperative normal thyroid function receiving PTX were enrolled for analysis from Department of Endocrine and Breast Surgery, the First Affiliated Hospital of Chongqing Medical University, Chongqing, China, from January 2017 to January 2019 (Table 1). This study was approved by the Ethics Committee of the First Affiliated Hospital of Chongqing Medical University. The inclusion criteria include that SHPT patients received parathyroidectomy + auto-transplantation under the general anesthesia and all enrolled patients have been assured with the normal thyroid function, normal thyroglobulin (Tg) and normal thyroid antibodies (such as thyrotrophin receptor antibody, TRAb; thyroid peroxidase antibody, TPOAb; anti-thyroglobulin antibodies, TGAb) before surgery. The exclusion criteria were those patients with previously known thyroid dysfunction (such as thyroiditis, hyperthyroidism), preoperative thyroid medication, prior thyroid surgery or parathyroid surgery.

**Table 1:** Characteristics of all patients (*n* = 24) undergoing parathyroidectomy plus auto-transplantation

Characteristics	Values
Number (female/male)	10/14
Age (year)	47.5 ± 11.9
Duration of dialysis (year)	8.0 ± 4.4
Operation time (min)	97.1 ± 27.1
Preoperative PTH (pg/ml)	2003.8 ± 998.3

*PTH* parathyroid hormone

### Blood biological studies

We assessed thyroid biochemical indicators in the morning before surgery and on day 1, 3 and 5 after surgery to compare changes in thyroid function. The biochemical indicators include Tg, high sensitivity thyrotropin stimulating hormone (sTSH), triiodothyronine (T3), free triiodothyronine (fT3), thyroxine (T4) and free thyroxine (fT4). Thyrotoxicosis was biochemically defined as serum sTSH below the normal minimum as well as fT4 above the normal maximum [13]. Additionally, we also need to assess the thyroid antibodies (such as TRAb, TPOAb, TGAb) in the morning before and after surgery to exclude out their abnormal changes.

### Statistical methods

The levels of thyroid biochemical indicators are displayed in the form of mean ± standard deviation. The SPSS software (Version 22.0) was used to analyze differences of the biochemical indicators before and after surgery by paired sample *T* test. *P* value of less than 0.05 was represented statistically significant.

## Result

**Fig. 1:**
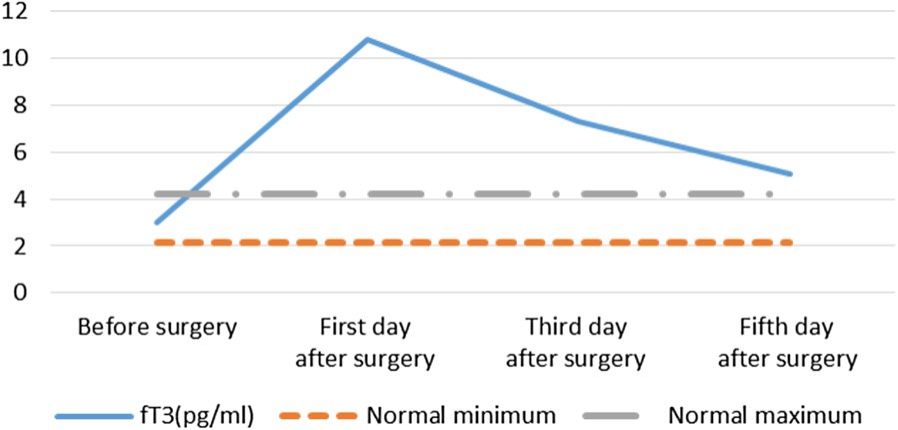
Fluctuation of mean value of fT3 in all patients (*n* = 24) before and on day 1, 3 and 5 after surgery. *fT3* free triiodothyronine

**Fig. 2:**
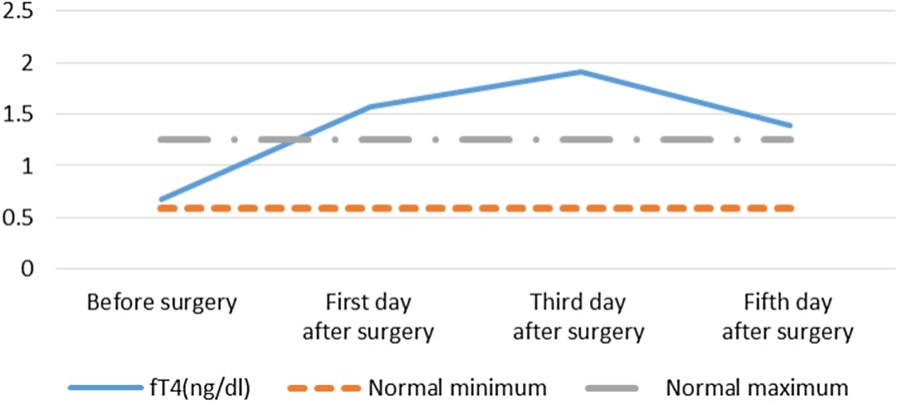
Fluctuation of mean value of fT4 in all patients (*n* = 24) before and on day 1, 3 and 5 after surgery. *fT4* free thyroxine

Among the 24 SHPT patients, 1 case (4.2%), 8 cases (33.3%) and 13 cases (54.2%) suffered thyrotoxicosis at the first, third and fifth day after surgery, respectively (Table 2). Serum FT3 level increased significantly (>2.5 times higher than the normal maximum) from pre-operation (3.00 ± 0.41 pg/ml, normal range 2.14–4.21 pg/ml) to the first day after operation (10.82 ± 7.22 pg/ml, p<0.001) and then gradually decline, but still higher than the normal maximum on day 3 (7.34 ± 4.63 pg/ml) and day 5 (5.10 ± 2.70 pg/ml) after surgery, respectively (Table 3 and Fig. 1). Serum FT4 level increased significantly (>1.5 times higher than the normal maximum) from pre-operation (0.68 ± 0.15 ng/dl, normal range 0.59–1.25 ng/dl) to the third day after operation (1.91 ± 0.97 ng/dl, p<0.001) and then gradually decline, but still higher than the normal maximum on day 5 after surgery (1.39 ± 0.58 ng/dl, p<0.001) (Table 3 and Fig. 2).

**Table 2:** Frequency of biochemical thyrotoxicosis (sTSH below normal level and FT4 above normal level) in all patients (*n *= 24), of which 17 patients with complete Tg data

	First day after surgery	Third day after surgery	Fifth day after surgery
T3↑	13 (51.2)	11 (45.8)	5 (20.8)
FT3↑	20 (83.3)	19 (79.1)	15 (62.5)
T4↑	13 (51.2)	14 (58.3)	8 (33.3)
FT4↓	13 (51.2)	20 (83.3)	13 (51.2)
sTSH↓	2 (8.3)	10 (41.7)	16 (66.7)
sTSH↓ and FT4↑	1 (4.2)	8 (33.3)	13 (54.2)
Tg	17 (100)	14 (82.4)	6 (35.3)

*T3* triiodothyronine, *T4* thyroxine, *fT3* free triiodothyronine, *fT4* free thyroxine, *sTSH* high sensitivity thyrotropin stimulating hormone

↑ Biochemical parameter above normal maximum

↓ Biochemical parameter below normal minimum. Frequency (percent)

**Table 3:** Patients' characteristics of thyroid function of all patients (*n *= 24) before and the days from surgery are displayed

Variables	Before surgery	First day after surgery	Third day after surgery	Fifth day after surgery
T3 (0.66–1.61 ng/ml)	0.98 ± 0.23	1.62 ± 0.74**	1.23 ± 0.52^†^	1.02 ± 0.42
T4 (5.44–11.85 µg/dl)	6.27 ± 1.71	11.59 ± 3.70**	12.67 ± 3.62^††^	9.74 ± 2.61^§§^
fT3 (2.14–4.21 pg/ml)	3.00 ± 0.41	10.82 ± 7.22**	7.34 ± 4.63^††^	5.10 ± 2.70^§^
fT4 (0.59–1.25 ng/dl)	0.68 ± 0.15	1.57 ± 0.78**	1.91 ± 0.97^††^	1.39 ± 0.58^§§^
sTSH (0.49–4.91 µIU/ml)	2.17 ± 1.09	1.52 ± 0.85*	0.92 ± 1.30^†^	1.12 ± 2.47
Tg (0.00–50.03 ng/ml)	9.93 ± 6.55	242.13 ± 154.05**	150.70 ± 132.08^††^	58.79 ± 46.30^§§^

Mean values and their standard deviation are given

*T3* triiodothyronine, *T4* thyroxine, *fT3* free triiodothyronine, *fT4* free thyroxine, *sTSH* high sensitivity thyrotropin stimulating hormone, *Tg* thyroglobulin

* Significant statistic difference between before surgery and one day from surgery (*P* value<0.05)

** Indicates *P *< 0.001

^†^Significant statistic difference between before surgery and 3 days from surgery (*P *< 0.05)

^††^Indicates *P *< 0.001

^§^Statistic difference between before surgery and 5 days from surgery (*P *< 0.05)

^§§^Indicates *P *< 0.001

**Fig. 3:**
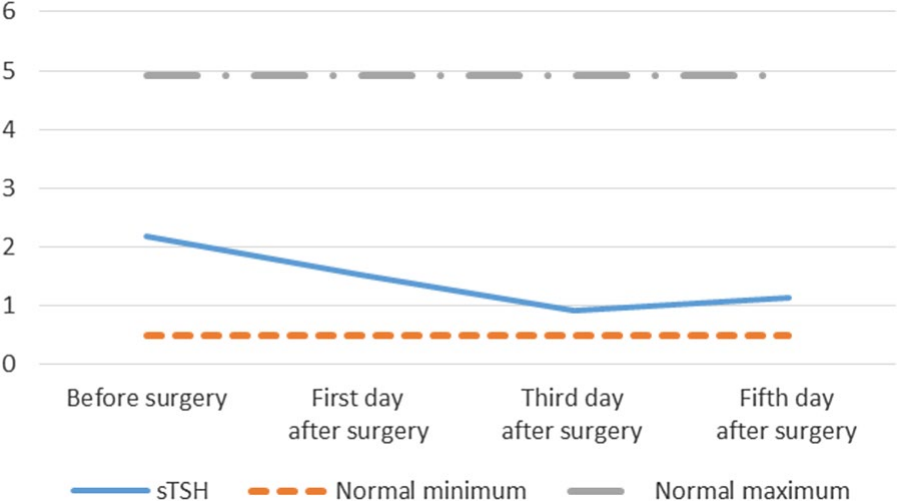
Fluctuation of mean value of sTSH in all patients (*n* = 24) before and on day 1, 3 and 5 after surgery. *sTSH* high sensitivity thyrotropin stimulating hormone

The mean value of serum sTSH always fluctuated within the normal range (Table 3 and Fig. 3), but the frequencies of serum sTSH lower than the normal level gradually increased from the first day (8.3%) to fifth day (66.7%) after surgery (Table 2). A total of 17 patients with complete Tg data, all of which were higher than normal maximum on the first day after surgery (Table 2), and the serum Tg level increased significantly (>4.8 times higher than the normal maximum) from pre-operation (9.93 ± 6.55, normal range 0.00–50.03 ng/ml) to the first day after operation (242.13 ± 154.05, *p<*0.001) and then gradually decline, but still relatively higher than the normal maximum on day 5 after surgery (58.79 ± 46.30 ng/ml, *p*<0.001) (Table 3).

## Discussion

In our center, we usually perform total parathyroidectomy ± auto-transplantation for the dialysis patients who have persistent secondary hyperparathyroidism (PTH levels > 800 pg/mL) and are accompanied by clearly related signs and symptoms and refractory to medical therapies. In the previous study, we found that thyrotoxicosis was an important postoperative complication of parathyroidectomy for SHPT patients [12], while only a few studies have focused on thyrotoxicosis as a result of PTX in patients with SHPT [11, 12]. That's why we conducted this retrospective study.

In this study, all the SHPT patients have normal thyroid function, normal Tg and normal thyroid antibodies (such as TRAb, TPOAb and TGAb) and have no preoperative thyroid medication, prior thyroid surgery or parathyroid surgery. It shows the common occurrence of thyrotoxicosis in these SHPT patients after surgery, 1 case (4.2%), 8 cases (33.3%) and 13 cases (54.2%) suffered thyrotoxicosis at first, third and fifth day after surgery, respectively (Table 2), suggesting that thyrotoxicosis was a common postoperative complication of parathyroidectomy for SHPT patients. All biochemical indicators of thyroid function have changed significantly after surgery. The frequencies of serum fT4 higher than the normal level are (51.2%), (83.3%) and (51.2%), on the first, third and fifth day after surgery, respectively (Table 2). The frequencies of serum sTSH lower than the normal level gradually increased from the first day (8.3%) to fifth day (66.7%) after surgery (Table 2). It indicates that thyroid hormone is abnormally released from the first day after surgery and the change of serum sTSH is later than that of fT4. This may be due to the massive release of thyroid hormone, and need time to inhibit the secretion of sTSH.

Research has shown that Tg is a good marker to evaluate the palpation thyroiditis [14]. Similarly, our study also showed that, there are 17 in the patients with complete thyroglobulin data, all of the Tg levels were higher than the normal maximum on the first day after surgery (Table 2). Thyrotoxicosis is caused by excessive circulating thyroid hormones for some reason. The common reasons include excessive production by the thyroid gland (as in Graves' disease), excessive production outside the thyroid, or loss of storage function and leakage from the gland [15]. The most likely reason for this study is mechanical insult during the surgery which causes transient loss of storage function. In SHPT patients with ESRD, parathyroid glands have long been stimulated by hyperphosphatemia and hypocalcemia, which leads to a tighter adhesion of the parathyroid glands to the thyroid gland. As a result, when clinicians are dissecting the parathyroid glands, it may inevitably stimulate and cause mechanical damage of the thyroid gland, resulting in a large amount of thyroid hormone releasing into the blood circulation, and leading to the occurrence of transient thyrotoxicosis. Thyrotoxicosis affects many different important organs, including neuromuscular, cardiovascular, pulmonary, gastrointestinal and skin [16, 17]. Some case reports have reported symptoms such as increased metabolism and atrial fibrillation in patients with thyrotoxicosis after surgery of SHPT, mainly manifested as tachycardia, palpitations, chest tightness, sweating and heat resistance and used to wear thin clothes and disliked sleeping covered with quilt [11, 12]. When thyrotoxicosis occurs, strengthen hemodialysis was effective measures to eliminate the excessive thyroid hormones, a beta blocker to control the symptoms and signs, bile acid sequestrants may also be of benefit in severe cases to decrease enterohepatic recycling of thyroid hormones, and glucocorticoids can be used to reduce T4-to-T3 conversion and promote vasomotor stability [15]. Besides, some traditional Chinese medicine might be helpful for thyrotoxicosis, such as Hydrangea paniculata stem and skimmin [18, 19]. All the principles are based upon clinical experience and case studies since there are no prospective studies.

Although the sample size is the largest in Asia, it is still small. A larger sample size is needed in the future to assess the incidence of thyrotoxicosis after surgery for SHPT with ESRD and normal thyroid function. The incidence of thyrotoxicosis may still be underestimated. One of the most important reasons is that we have predictive dialysis to improve patient prognosis and reduce serum thyroid hormone levels indirectly.

In conclusion, most of the SHPT patients with ESRD and normal thyroid function are likely to suffer thyrotoxicosis after parathyroidectomy. Clinicians should pay attention to this common complication, and evaluate the thyroid function, especially FT3, FT4 and sTSH, and the relating symptoms in these patients during perioperative period to make early diagnosis and appropriate prevention and treatment of thyrotoxicosis.

## Data Availability

The datasets used and/or analyzed during the current study are available from the corresponding author on reasonable request.
